# Hospital Anticipatory Care Planning for Inpatients of Organic Old Age Psychiatry Wards (NHS Lanarkshire)

**DOI:** 10.1192/bjo.2021.480

**Published:** 2021-06-18

**Authors:** Sarah Brennan, Rajdeep Routh

**Affiliations:** NHS Lanarkshire

## Abstract

**Aims:**

To improve practice of Hospital Anticipatory Care Planning for inpatients of Organic Old Age Psychiatry wards in NHS Lanarkshire.

**Background:**

Hospital Anticipatory Care Plans (HACPs) are important components of care for inpatients with progressive and life-limiting conditions. HACPs provide guidance on treatment escalation and limitation for individual patients, in the event that they become acutely unwell. In the Old Age Psychiatry Department at NHS Lanarkshire, HACP standards are as follows:

- HACP forms should be completed within 2 weeks of admission
- HACP information leaflets should be provided to relatives/carers
- HACPs should be discussed with relatives/carers
- If a patient without an HACP becomes acutely unwell, an HACP should be made, and the responsible Consultant informed
- HACP should be discussed within the multi-disciplinary team (MDT)
- HACPs should be regularly reviewed
- HACP and DNACPR forms should be kept at the front of the notes
- Superseded HACPs should be marked as obsolete

**Method:**

Inpatient notes were reviewed in October 2019 and compared against the above standards.

The findings were presented at the Clinical Governance Meeting and Old Age Psychiatry Teaching Group in December 2019.

An ‘HACP Checklist’ was also created to prompt good practice.

Inpatient notes were reviewed again in July 2020.

Data from both time periods were compared.

**Result:**

There was an improvement in:

The proportion of patients who had an HACP - from 59% to 96%

The proportion of patients who had an HACP made within 2 weeks of admission - from 35% to 78%

Documentation of HACP discussions with relatives/carers - documented for 77% of patients (from 47%)

Timing of HACP discussions with relatives/carers - took place within 2 weeks for 52% of patients (from 29%)

Documentation of HACP discussion by MDT - documented for 73% of patients (from 29%)

HACP Information Leaflets were only distributed to one patient's relatives/carers across both time points

Medical emergencies for patients with no HACP were infrequent and so comparison could not be made

HACPs were reviewed less frequently in July 2020 than in October 2019

HACP forms and DNACPR forms were always filed appropriately

Superseded HACP forms were always appropriately marked as obsolete

**Conclusion:**

HACP practice mostly improved from October 2019 to July 2020. This may have been due to increased awareness of HACP Standards, following the presentation of initial data to inpatient teams.

A much larger influence, however, was likely to be the COVID-19 pandemic and associated efforts to improve HACP practice throughout the Health Board.

